# Relationships between job characteristics and occupational well-being: Are they similar across levels of analysis?

**DOI:** 10.1371/journal.pone.0328508

**Published:** 2025-07-24

**Authors:** Marc van Veldhoven, Riccardo Peccei, Aneeqa Suhail, Karina van de Voorde, Marcel Croon, Joran Jongerling

**Affiliations:** 1 Department of HR Studies, Tilburg University, Tilburg, Netherlands; 2 Department of Management, King’s College London, London, United Kingdom; 3 Department of Statistics and Methodology, Tilburg University, Tilburg, Netherlands; Guangxi Normal University, CHINA

## Abstract

Theory and practice in occupational health psychology have hitherto mostly assumed that how job characteristics relate to occupational well-being is similar across levels of analysis, yet this remains empirically underexplored. We tested this implicit “assumption of homology” using the Demand-Control Model as our starting point. We analyzed three-level data from 12,658 employees in 1,116 work units from 243 organizations in the Netherlands. Results indicate that for job demands and participation homology of relationships is mostly confirmed, but not for skill variety and job autonomy. In addition, we generally did not find relationships becoming stronger from the individual to the departmental to the organizational level. Future theory needs to conceptualize better how individual and aggregate-level effects of skill variety/autonomy combine and interact in influencing occupational well-being. For these job characteristics we need multi-level theorizing. For practice, our results point towards caution in using individual survey scores on skill variety/autonomy for the purpose of risk monitoring and proposing follow-up policy/interventions at aggregate levels such as departments and organizations.

## Introduction

Job characteristics and occupational well-being are important issues in applied psychology, particularly amid ongoing organizational and technological change in the workplace. Monitoring occupational well-being and its psychosocial risk factors has become common practice at various levels, including work units, organizations, industries, and countries [1]. This type of monitoring informs occupational health initiatives targeted at managing work-related stress, well-being and performance [2]. However, most research on how job characteristics impact occupational well-being relies on individual-level survey data [3], while policy and intervention implications often target the supra-individual levels (e.g., departments, job groups, organizations as a whole, branches of industry, and countries [4]). Such follow-up initiatives are based on the assumption that the relationship between job characteristics and occupational well-being is similar at the individual and supra-individual levels.

Additionally, as part of an attempt to gain a better multi-level understanding of job characteristics and well-being, there has been a growing focus on how the organizational context shapes work design processes [5]. Researchers have suggested that a trickle-down effect may be in place [6], where characteristics at the organizational level (like climate and HR practices) may influence team-level processes of job demands/resources and well-being, which in turn influence individual-level processes of job demands/resources and well-being. For example, the study by Croon et al. 2015 [7] demonstrated how job enrichment practices in workplaces translated into individual job satisfaction via improved job resources. Such a multi-level extension of theorizing on job characteristics and well-being, however, also assumes homology in the relationships under investigation across levels.

**Fig 1:**
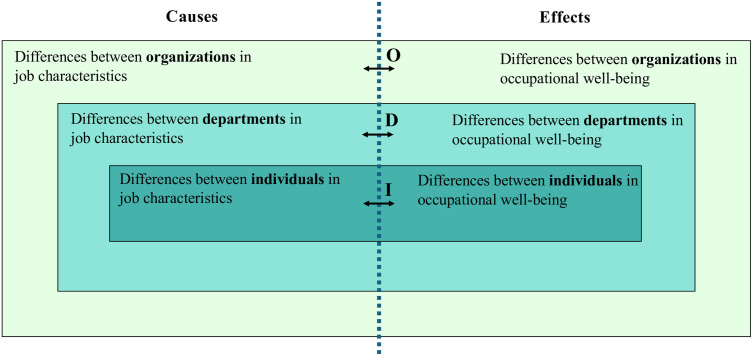
Homology of relationships across three levels. The assumption of homology implies that cause-effect relationships I, D and O are the same across the three levels within the data structure of individual employees nested within departments nested within organizations.

Fig 1 further illustrates homology of relationships between job characteristics and occupational well-being. It highlights that individual employees are nested within departments, and these departments are, in turn, nested within organizations. Departments may vary in size, and organizations may contain different numbers of departments. Individual-level survey data is typically used to make statements about job characteristics and occupational well-being in departments and organizations, usually by reporting on sum or mean scores for departments and organizations. Findings as to sum scores/means in job characteristics are seen as causes of sum scores/means found in occupational well-being (effects). The implicit assumption here is that how differences between individual employees in job characteristics (causes) are related to differences in occupational well-being (effects) between individual employees (I), is similar to how differences in causes between departments are related to differences between departments in effects (D), and that differences in causes between organizations are similarly related to differences between organizations in effects (O). In short, “homology of relationships” means that the cause-effect relationships I, D, and O are comparable across the three levels.

To our knowledge, this assumption about homology (the terms equivalence and isomorphism are sometimes also used to denote this, see [8,9]) of relationships across levels has never been explicitly tested empirically. As we discuss more fully below, there have been only a few studies that have adopted a multi-level perspective to examine the link between job characteristics and occupational well-being across levels. The findings of these studies suggest that job characteristics may have different effects at the job/team/organizational versus individual levels [10–12]. If this is true, disentangling how job characteristics relate to occupational well-being at different levels is crucial for improving risk monitoring practices and advancing theoretical knowledge in occupational health psychology.

This study is designed to contribute to the occupational health literature by explicitly testing whether the “homology of relationships” assumption is actually met in survey data on job characteristics and occupational well-being. In particular, we use the Demand-Control Model as a basis for testing whether survey data on job characteristics and occupational well-being with a three-level nested structure (individuals within departments within organizations) confirm the assumption of relationship homology at different levels of analysis. The Demand-Control Model is widely accepted in research and practice on work, health, and performance [13,14]. It is an example of a situationistic/job characteristics approach to understanding occupational well-being, e.g., an approach that highlights how exposure to specific environmental risk factors in the work situation impacts on occupational well-being. This in contrast with approaches that emphasize factors outside the work situation, like recovery activities during leisure time, factors in the employee’s resilience or abilities (individual differences), or approaches that highlight interactions between factors, such as work-family balance. For an overview of various approaches, including a range of situationist approaches focusing solely on job characteristics, see the textbook by Peeters, De Jonge & Taris [15]. For an example of an alternative, more interactionistic approach, see [16]. Limiting our scope to the set of job characteristics figuring in the Demand-Control Model helps us to align our study with the risk monitoring approach that is common in practice and policy in occupational health psychology, as well as delimit our analysis to a restricted but manageable set of key job characteristics, thereby enabling us to focus on the homology of relationships issue that is at the centre of the study, rather then challenging the theoretical rationale of the model as such (which has been done elsewhere, see [17,18]).

To this end, we will address three research questions. First, we will explore to what extent individual-level, departmental-level, and organizational-level relationships involving key job characteristics and occupational well-being indicators are the same, e.g., whether homology of such relationships exists. Second, following Karasek’s (1979) Demand-Control Model ([19], we will test whether high job demands are consistently associated across levels with high job strain, and high job control with high levels of positive job attitudes. Third and finally, in this paper, we explore whether there is any indication of cause-effect relationships being stronger at aggregate levels than at the individual level, as one could argue that from individuals to departments to organizations the effect of measurement error in assessing job characteristics is reduced [20], and at aggregate levels the shared perceptions of the individuals working within a department and/or organization are measured [21].

### Job characteristics and employee well-being from a multi-level perspective

#### Shared perceptions of job characteristics.

In the Demand-Control Model, job demands and job control are considered to be features of the objective work environment that not only impact a single individual employee but that are expected to influence all incumbents of a particular job and/or all employees in a certain work unit, department, organization, branch of industry, country or any other relevant grouping variable [17,22]. This line of thinking is closely modelled after how occupational health and safety research theorizes the health consequences of physical risks in work environments [23].

As such, the Demand-Control Model is a situationist model, and one would thus expect a tendency towards measuring job demands and job control with objectifiable measures at the level of groups of workers sharing a similar job setting or work environment. However, the large majority of the research in this area actually uses individual survey measures [10,23]. This has been criticized by authors who wondered how situationist this line of academic research actually is [24–26].

It is common to answer to such criticism by relying on inter-subjectivity, let’s explain. When using individual survey data within a situationist framework like the Demand-Control Model, a shared component is assumed to exist in survey responses of employees who are exposed to a similar job setting and/or work environment [10,23]. Using the survey method on groups of employees to chart this shared component is thought to deliver valid and reliable measures of the external, objective exposure to environmental risk factors [27,28]. As a consequence, survey measures of job characteristics that are aggregated to the group level (sum scores/means) are assumed to provide more reliable and accurate indicators of the objective work environment than individual survey measures because the use of multiple raters for assessing the shared environment reduces measurement error (inter-subjectivity).

Several studies have empirically investigated the extent to which workers in units indeed demonstrate a shared component in their experiences of job demands and job control. Most notably, Morrison, Payne & Wall (2003) [29] reported a two-level study with 6,700 individual employees in 81 different jobs in the UK National Health Service. For job demands, these authors found 17% of shared variance in jobs, and for job control 25% of shared variance. Other studies, including [10,11,30,31] have also reported on such shared components. In these studies, shared variance for job demands ranged from 11% to 15%, while the range for job control was broader, spanning from 5% to 38%.

#### Shared occupational well-being experiences.

The Demand-Control model predicts that groups of workers exposed to high demands in combination with low control are at risk for reduced occupational well-being. A stream of literature offers conceptualizations that might help to understand why there might be shared consequences in terms of occupational well-being for workers sharing similar job characteristics. Related to attitudinal types of well-being, George (1990) [32] proposed the concept of group affective tone, which is defined as consistent or homogeneous affective reactions within a group. A construct that is close to group affective tone is that of group task satisfaction. This was put forward by Mason and Griffin (2002) [33], who defined it as a group’s shared attitude towards its task and the associated work environment.

More focused on health-related types of well-being Semmer, Zapf and Greif (1996) [22] have proposed the concept of “shared job strain”, which refers to that part of strain that different workers holding the same job have in common. These authors show in a sample of 932 German male blue-collar workers that the amount of variance explained in shared job-strain (e.g., shared by two incumbents for a job) is higher than that found in regular studies explaining individual job strain. In a similar vein, Cole, Bruch and Vogel (2012) [34] proposed the concept of collective energy. Collective energy is described as the shared experience and demonstration of positive affect, cognitive arousal, and agentic behavior among unit members in their joint pursuit of organizationally salient objectives. Finally, literature on team dynamics and processes has been used to conceptualize the emergence of team engagement and burnout, which refer to shared experiences of exhaustion and disengagement [35,36].

Turning to empirical research, several authors have reported shared variance in occupational well-being by workers in units. Using data from 254 salespeople in 26 groups, George (1990) [32] demonstrated a sufficient amount of within-group agreement to suggest that “group affective tone” indeed exists. In the study by Morrison, Payne, and Wall (2003) [29] the job category explains 5.4% of the variance in job satisfaction. Other studies have also reported on shared well-being [31,37,38], with findings ranging from 2% to 13%.

In summary, both theoretical and conceptual arguments support the presence of shared variance in work units regarding job characteristics as well as occupational well-being. The empirical evidence for shared variance in job characteristics appears to be somewhat stronger than for occupational well-being. In fact, the limited shared variance in occupational well-being measures is a restricting factor in any application using individual survey data for supra-individual level purposes of predicting, monitoring, and managing job-related strain and satisfaction, as Morrison, Payne, and Wall (2003) [29] made clear. We will nevertheless proceed in this paper while building on the assumption that shared, objective environmental demands and control characteristics in departments and organizations translate into reliable, shared experiences of these environmental features as measured by individual surveys among workers in these units/organizations. These, in turn, predict shared experiences of occupational well-being.

### Multi-level analyses connecting job characteristics to occupational well-being

Over time, multi-level analysis has entered into the arena of research examining the relationship between job characteristics and occupational well-being, particularly in studies related to the demand-control model. Most studies that were hitherto published have used a macro-micro perspective on multi-level analysis [39]. This means that independent variables (demands and control) derive from individual and supra-individual levels. Still, the dependent variable (occupational well-being) is analyzed at the individual employee level only [21]. Although pragmatically, this choice is entirely comprehensible, it does not inform our current research question, which seeks to understand the homology of relationships between psychosocial job characteristics and occupational well-being across different levels of analysis.

Only two earlier studies were found that examined how job characteristics at multiple levels relate to well-being at multiple levels: Van Veldhoven et al. (2002) [40] and Morrison, Payne & Wall (2003) [29]. The latter authors found that job-level shared variance in job satisfaction is explained to a large extent by independent variables relating to the expanded Demand-Control model. Notably, the R^2^ based on these independent variables is considerably higher at the job level (74%) than at the individual level (32%). In addition, the regression coefficients for job control in relation to job satisfaction are consistently positive, both for the individual and the job level (.26 and .19, respectively, in the final equation). For job demands, however, the individual level variable is negatively associated with job satisfaction (−.35 in the final equation), whereas the job level variable is positively associated with satisfaction (.16 in the final equation). Van Veldhoven et al. (2002) [40] also found that explained variance is higher at the aggregate level than at the individual level. Additionally, similar to Morrison, Payne and Wall (2003) [29], they found that individual-level job characteristics and aggregate-level job characteristics did not show consistently positive or negative relationships with occupational well-being.

According to these two studies, it is anticipated that relationships observed at aggregate levels of analysis (such as job categories, departments, organizations) will be stronger in comparison to those at the individual level of analysis. This is primarily because measurement error is supposed to be largest at the individual level due to individual variation in exposure, personality factors determining perceptions (of environment and self), and individual differences in response tendencies [41]. When moving from the individual to aggregate levels of demands, control and well-being measurement, reliability is expected to increase, and group means are expected to reflect shared situational conditions and their well-being implications better. This line of reasoning aligns well with the situationist roots of the Demand-Control Model [19].

## Method

The present study is based on data derived from a cumulative database of the Questionnaire on the Experience and Evaluation of Work (QEEW) used in the Netherlands [28,41].

### Context of data collection

For our research purpose, a nested data structure is necessary, which rules out National or International databases that tend to use random samples from the workforce. As such we use nested data that is derived from a large-scale collaborative project by Dutch occupational health care services. The individual occupational health care services conducted survey projects on job characteristics and occupational well-being in organizations on a one-by-one basis but agreed to aggregate the surveys in a central database for the purpose of building reference data and doing applied research. More details about the data collection context are reported in Van Veldhoven & Broersen (2003) [28]. For the current study, we revisited the archival data set of that paper. At the time of data collection informed consent was not a common practice in Dutch occupational health care research. For the purposes of building reference materials and applied research, all variables that would allow the identification of individual employees, departments and organizations were removed from the archival dataset. Participation by individual workers was always voluntary. Response rates per project typically varied from 60% to 80% in this research context.

For the current study, we received approval from the Ethical Review Board at the Tilburg School of Social and Behavioral Sciences (TSB_RP1766). The secondary analyses were performed in various stages between March and October 2023. Although the nesting structure of the data was preserved in the archive, the authors had no access to information that could in any way trace the data to specific individuals, departments, or organizations that participated.

### Data selection

We used a selection of data from the central database, namely 12,658 shopfloor employees working in 1,116 departments within 243 organizations. The cases selected comply with the following set of criteria: (1) full data were available for Warr’s (1990) [42] occupational job anxiety-contentment scale, and (2) respondents were non-managerial, shop-floor employees from departments with 5–25 respondents. The first criterion is directly related to the purpose of the present study and is the most important criterion in terms of data availability. Warr’s (1990) [42] scale is part of the extended QEEW-questionnaire and was only administered in 1 out of 5 projects. The second criterion deserves some further elaboration.

We aimed to create optimal conditions for researching the current research question, e.g., homology. For this purpose, it was important for the departments involved to be neither too small (potential disproportionate influence of individual (extreme) responses as opposed to more structural group-based components) nor too big (potential fuzzy composition of the department in terms of job characteristics). In addition we decided to only include shopfloor employees, managers were excluded. This approach was taken to ensure that department-level variation in scale scores accurately represents a structural, group-level component, untainted by potential biases that might arise from managers’ survey responses, and/or the fact that management roles likely differ from shopfloor jobs within a department.

The response rates among employees were high, suggesting that non-reponse is unlikely to have extensively biased the results of this study. At the organization level, we do not claim that the current data are representative of all organizations in the Netherlands. However, the organizations participating in the study encompass a wide range of industries, and are typical of the clientele served by occupational health care services in the Netherlands.

The database used for this study not only has a nested data structure, it also has sufficient numbers of cases at all three levels investigated. This sets the dataset apart from most other multi-level datasets used in the literature to investigate the demand-control model in relation to occupational well-being that were cited before. A copy of the selected datafile and a codebook describing its contents are available on the journal’s website.

### Demand-control scales

Job demands were measured with the QEEW-scale *Work speed and quantity.* This scale exclusively focuses on quantitative aspects of workload in terms of required speed and amount of work. Questions from the psychological work demands scale by Karasek (1985) [40] were used as a guideline in developing this scale. Respondents are asked to answer 11 items with four answering categories in terms of frequency (always, often, sometimes, never). A negative sample item is “Do you work under time pressure?”. A positive sample item is “Can you do your work at your ease?”. Cronbach’s alpha in the current sample is .88.

In this study, we covered both aspects that Karasek (1985) [43] included in his original job control measure. He referred to the combination of the two aspects as ‘decision latitude’. The two elements are skill discretion and task autonomy. These two are measured here with the task autonomy and skill variety scales from the QEEW. These two scales were developed based on the well-known scales by Karasek (1985) [43] and by Hackman & Oldham (1975) [44]. The scales use a 4-point response format (always, often, sometimes, never) as answering categories. The *(lack of) task autonomy* scale consists of 10 items on the level of control an employee has over timing and work method. Sample items are: “Do you have an influence on the pace of your work?” and “Can you decide on how your work is executed?”. Cronbach’s alpha is .90. The *(lack of) skill variety* scale has 6 items on employees’ experience of job variety, opportunities for creativity and skill use in their job. Sample items are: “In your work, do you repeatedly have to do the same things?” (negative item) and “Does your work require creativity?” (positive item). Cronbach’s alpha is .82.

In the context of the job design literature, skill variety and task autonomy are the most recognized features of job control [45]. In the literature on labor relations/ industrial relations, however, the social or political side of job control is considered equally important. This feature is recognized by Karasek and Theorell (1990) [27], in their discussion on the Quality of Work Life/Industrial Democracy movement. Therefore, it seems relevant to include this element of job control in our study as well, particularly from the perspective of possible job features that could be shared by employees within organizations. Hence, a QEEW-scale called *(lack of) participation in decision-making* was included in the study. This 8-item scale focuses on job control aspects at the collective level (as opposed to the task level). The answering categories range from 1 = *never* to 4 = *always*. The instrument comprises different control aspects, such as the ability to discuss problems with supervisors, the amount of influence in the work group, and involvement in group decision-making. Cronbach’s alpha is .87.

### Occupational well-being scales

We have chosen to use three separate scales, each measuring different facets of the occupational well-being construct (attitudinal as well as strain-related types of well-being).

The first scale selected was *(lack of) task satisfaction.* This 9-item dichotomous scale (yes/no) can be interpreted as the opposite of work pleasure. Most of the questions are formulated in ways that inquire about feelings of task resistance (on the negative side) or task engagement (on the positive side). Such items have been shown to be reflective of the task and work environment of groups of workers by Mason & Griffiin (2002) [33], and to complement the literature on general job satisfaction [46,47]. Sample items include “I find my work stimulating, each and every day” (positive) and “I have to continually overcome resistance in order to do my work” (negative). Cronbach’s alpha is .79.

The second scale concerns *need for recovery.* Need for recovery relates to strain accumulation and is based on the Effort-Recovery Model [48]. Within the normal effort-recovery cycle “need for recovery” is characterized by temporary feelings of overload, irritability, social withdrawal, lack of energy for new effort, and reduced performance. A scale was developed in the QEEW to measure the need for recovery. It contains 11 statements, and the respondent is asked to rate whether these statements apply using a yes/no-format. A sample of a negative item is: “Generally, I need more than an hour before I feel completely recuperated after work”. A sample of a positive item is: “After the evening meal, I generally feel in good shape”. For the sample in this study, we found a Cronbach’s alpha of .88.

The last scale selected is based on the *job-related anxiety-contentment* scale [42]. This scale measures one of the three dimensions in Warr’s framework of occupational well-being, specifically the level of contentment as opposed to anxiety experienced on-the-job. Employees are asked to report to what extent they experienced six emotions during the past working week on a 4-point scale (completely not, hardly, somewhat, completely). For a fuller account of this scale, the reader is referred to Warr (1990) [42], and Sevastos, Smith and Cordery (1992) [49]. The Cronbach’s alpha is .62. High scores refer to higher anxiety and less contentment, and for purposes of simplicity, we’ll henceforth use the label “job anxiety”.

### Analysis

Statistical analyses in the context of research into the homology of theoretical constructs and their relationships should concentrate on how the association among these constructs varies at the different levels of analysis. In the present study, three levels are distinguished: organizations, departments within organizations, and individuals within departments. To analyse the relationships between the constructs on the higher (i.e., department and organizational) levels, we do not use (weighted) aggregation to group means as is done in other studies [27]. The reason for this is that using observed sample group means biases results [7,50–54], as the variances and covariances between these observed aggregated means not only represent true variation at that level but also reflect variation at any of the lower levels. Therefore, instead of calculating these group means, we will include them as latent variables in our model and estimate them along with the other parameters. This approach is called latent mean centering and has been shown to avoid the biases associated with observed score aggregation. It is integrated into the software package Mplus [54], where it is the default method for multilevel analyses. For the current analysis, we modelled all three levels in one model and used Bayesian estimation.

### Checking variance and reliability

To investigate whether there is a significant amount of variance and adequate reliability for all levels intended for analysis, we present the ICC1 and ICC2 statistics as proposed by Bliese [55].

### Testing relationships at different levels of analysis

In order to examine whether the relationships between the measurement scales are consistent across different levels, three derived correlation matrices were obtained using the scale scores rather than the item scores. The correlation structure between the scales can be understood by inspecting the (bivariate) correlations. However, since some of the scales can be considered as dependent variables that are possibly “explained” by the remaining independent variables, a series of multiple linear regression analyses is carried out at each level to elucidate the relationships between dependent and independent variables.

The Mplus-script used to perform these analyses is included in the Appendix 1 in S1 File. In assessing the relationships of job demands and job control with occupational well-being we have not investigated possible interaction effects. The reason for this is the somewhat mixed results that have been reported in the literature for such proposed interaction effects [13,56].

## Results

### Shared variance at the supra-individual levels

We started our analysis by checking to what extent our data meets the statistical requirements for the intended analysis. In Table 1, we first present the ICC1 for our scales, taking the three-level data structure into account.

**Table 1:** ICC1 and ICC2 at the level of departments/ organizations, considering three levels simultaneously.

Scale	Departments		Organizations	
	ICC1	ICC2	ICC1	ICC2
Job demands	.12	.70	.04	.80
Lack of skill variety	.11	.76	.12	.89
Lack of task autonomy	.10	.77	.13	.90
Lack of participation	.08	.66	.07	.83
Lack of task satisfaction	.02	.42	.04	.71
Need for recovery	.03	.48	.03	.70
Job anxiety	.03	.41	.02	.63

The figures at the department level range between 8% and 12% for job characteristics and between 2% and 4% for occupational well-being. At the organizational level, we find values between 4% and 13% for job characteristics and between 2% and 4% for occupational well-being. These results are all highly significant, e.g., the grouping variable consistently explains significant amounts of shared variance (ICC1).

A second set of results concerns the ICC2 values, i.e., the reliability of the comparisons between units at a certain level. For the individual level, this amounts to Cronbach’s alpha, which was adequate for all scales used. Table 1 shows that ICC2 for the job characteristics is always above .70. The exception to the rule (lack of participation) is just below .70. For well-being at the organizational level, ICC2 values are around.70 as well. However, at the department level, values for ICC2 fall below .50 for occupational well-being. They are close to the minimum requirement (e.g., .40), but only marginally adequate. Results at the department level, therefore, need to be interpreted with some caution.

### Correlations between job characteristics and occupational well-being at the three levels of analysis

The correlation matrices for each of the three separate levels of analysis are reported in Table 2. Although some correlations show a pattern of increase from individual to department to organization level, this pattern was not found for all correlations. It is worthwhile to note that demands and lack of participation are positively related at all levels, indicating that more opportunities for participation are linked to fewer demands. The correlation between lack of skill variety and demands, however, is weak and negative at all three levels. Moreover, for lack of autonomy, the positive correlation coefficient decreases from the individual to the department to the organizational level, suggesting that lower job control in terms of autonomy is associated with higher demands. Low control in terms of skill variety, however, aligns with low demands, especially at the department level.

**Table 2:** Correlation matrix at three levels.

		1	2	3	4	5	6
**Scale**	**Level**						
**1. Job demands**	**Ind**						
	**Dept**						
	**Org**						
**2. Lack of skill variety**	**Ind**	−.09***					
	**Dept**	−.16***					
	**Org**	−.01					
**3. Lack of task autonomy**	**Ind**	.20***	.28***				
	**Dept**	.09**	.51***				
	**Org**	.06	.55***				
**4. Lack of participation**	**Ind**	.10***	.33***	.45**			
	**Dept**	.16***	.52***	.65***			
	**Org**	.23**	.73***	.60***			
**5. Lack of task satisfaction**	**Ind**	.13***	.33***	.18***	.30***		
	**Dept**	.20***	.49***	.33***	.50***		
	**Org**	.25**	.34***	.13*	.56***		
**6. Need for recovery**	**Ind**	.41***	.05***	.17***	.19***	.31***	
	**Dept**	.72***	−.14**	.26***	.19***	.46***	
	**Org**	.45***	−.04	.19**	.21**	.35***	
**7. Job anxiety**	**Ind**	.41***	.09***	.23***	.27***	.37***	.51***
	**Dept**	.73***	−.16***	.09	.27***	.50***	.72***
	**Org**	.52***	−.27***	−.23**	.23**	.54***	.59***

Abbreviations: Ind: individual level; Dept: departmental level; Org: organizational level.

N (ind)=12,658, N (dept)= 1,116 and N(org)= 243. Standardized regression coefficients are reported.

p < .1, * p < .05, ** p < .01, ***.

### Relationships between job characteristics and occupational well-being

In Table 3, the multiple regression results are reported for the unbiased estimates of the relationships between job characteristics and occupational well-being. In sum, this Table confirms the homology of relationships for job demands and lack of participation. For lack of skill variety and task autonomy, we find positive as well as negative betas, which does not confirm the homology of relationships.

**Table 3:** Relationships between job characteristics and occupational well-being.

	Lack of task satisfaction			Need for recovery			Job anxiety		
	Ind	Dept	Org	Ind	Dept	Org	Ind	Dept	Org
**Job demands**	14***	.22***	.09	.40***	66***	.36**	.38***	.66***	.29**
**Lack of skill variety**	.28***	.39***	−.05	.04***	−.19**	−.36	.04***	−.19**	−.77***
**Lack of task autonomy**	−.03**	−.09	−.32***	.01	.28***	.22	.05***	−.10	−.43***
**Lack of participation**	.21***	.32**	.78***	.13***	.02	.25	.13***	.35**	.97***
Adjusted R-square	.17***	.36***	.41***	.19***	.59***	.28***	.22***	.62**	.67***

Abbreviations: Ind: individual level; Dept: departmental level; Org: organizational level.

N (ind)=12,658, N (dept)= 1,116 and N(org)= 243. Standardized regression coefficients are reported.

p < .1, * p < .05, ** p < .01, ***.

First, for job demands, the beta coefficients are positive and significant, except for the relationship with (lack of) task satisfaction at the organizational level. Also, in relation to job demands, the betas are higher for need for recovery and job anxiety than for lack of task satisfaction, and the highest beta values are found at the department level. Second, for (lack of) participation, all estimates are positive. Here, for need for recovery, we find lower estimates than for the other two well-being indicators. The highest beta values are found at the organizational level. We also found that demands are more strongly associated with need for recovery/job anxiety than lack of task satisfaction. Lack of participation is more strongly related with lack of task satisfaction than with need for recovery, but we also see strong associations with job anxiety at aggregate levels. For both job demands and lack of participation, betas tend to be higher at aggregate levels than at the individual level.

Third, for (lack of) skill variety, we find that for lack of task satisfaction the demand-control predictions hold at individual/departmental level, but for the strain indicators, betas are low (individual level) or negative (department/organization level). Fourth, we also find quite a lot of negative betas for (lack of) task autonomy. This concerns 5 out of 9 coefficients (not all of which are significant). Most of these betas are not very sizeable, but at the organizational level, there are moderately significant negative betas between (lack of) task autonomy and (lack of) task satisfaction, as well as between (lack of) task autonomy and job anxiety. Finally, there is some tendency for betas to be higher at aggregate levels than the individual level where the relationships of lack of autonomy/skill variety and need for recovery/job anxiety are concerned (even though the signs are not always as expected). This is not the case for lack of skill variety in relation to lack of task satisfaction, however, where the beta at the individual level is clearly higher than at the organizational level.

Turning to the R^2^-values for the four Demand Control variables simultaneously, we find that between 17% and 67% of the variance in occupational well-being is explained by the four variables. At the aggregate levels, the R^2^-values are higher than at the individual level. Indeed, some of the beta coefficients become very high at aggregate levels.

## Discussion

The results of this study highlight three important substantive points regarding homology in the relationships between job demands and occupational well-being across levels.

Firstly, we confirmed that there is homology of relationships between job demands and occupational well-being. Similarly, confirmation was found regarding lack of participation and occupational well-being. However, for lack of skill variety and lack of autonomy the results did not completely support the idea of homology in relationships with occupational well-being. In this context, it is useful to distinguish between full and partial homology. Full homology refers to situations where specific job characteristics to well-being relationships are consistent across all three levels, while partial homology refers to situations where equivalent relationships are only present at two of the three levels. Our results show that the extent of full homology in our data was limited, particularly when examining the relationship between job characteristics and well-being in line with the theoretical predictions by the Demand-Control model. Thus, only four out of the 12 potentially homologous relationships fitted the pattern completely, based on the significance of the regression coefficients. In two cases, however, the relationship at one of the three levels, although significant, was quite weak (below .30 or above −.30, see [57]). In the end, therefore, only two of the 12 relationships examined, namely those between job demands and both strain-related well-being indicators, were found to be at least moderately strong, to be positive, and to reproduce at all three levels of analysis (full homology). Partial homology was found in the other two instances, e.g., moderately strong, positive relations were found only for two of the three levels. Both concern (lack of) participation, connected with either (lack of) task satisfaction or job anxiety. In the other eight instances, we did not observe homology of relationships.

Secondly, we discuss how our results confirm the specific predictions by the Karasek [19] model. At each level, we examined the relationship between job demands and three indicators of lack of job control (i.e., lack of skill variety, lack of task autonomy, and lack of participation) and three indicators of lack of well-being at work (i.e., lack of task satisfaction, need for recovery, and job anxiety). In total, therefore, we examined 12 main job characteristics-well-being relationships per level. The results of the multivariate analysis revealed that 10 out of the 12 relationships at the individual level were in line with the Demand-Control model predictions. However, eight of these relationships were quite weak (below .30 or above −.30, see [54]), indicating only limited support for the notion of a systematic connection between these job characteristics and employee well-being at the individual level. At the individual level, the strongest association was between job demands and strain-related aspects of well-being, e.g., specifically with need for recovery and job anxiety.

A different pattern of results emerged at both the department and organizational levels. At the departmental level, seven of the 12 relationships are in line with our theorizing, with only two relationships showing weak correlations. Again, at the department level, the strongest association was between job demands and the various strain-related measures of well-being. In addition, though, at the department level, two indicators of well-being were found to be moderately related to participation. Furthermore, we found some negative relationships, specifically between skill variety and the strain-related indicators of well-being, which do not support the Karasek (1979) [19] model. Finally, at the organizational level, only four of the 12 relationships were consistent with Karasek’s original theory. The strongest positive relationship at this level was between participation and two indicators of well-being. Job demands were also positively associated with the two strain-related well-being indicators at this level. However, three relationships between task autonomy and skill variety, on the one hand, and well-being, on the other, were significant but negative, thereby contradicting the predictions of the Karasek (1979) [19] model.

The third, related but more general substantive point of discussion pertains to the situationist arguments underpinning the Demand-Control model. As noted by Morrison et al. (2003) [29] and Härenstam (2008) [23], although the Karasek model has been tested primarily at the individual level, the model was originally conceptualized at the job level. Employees in a specific job category were theorized to collectively differ in exposure to environmental conditions of job demands and job control, compared to employees in other job categories. By conceptualizing in terms of exposure, psychosocial job factors could easily be integrated into common frameworks for preventing and managing risks to health and safety in the work environment. If this underlying premise of the model is accurate, we would expect the stressor-strain relationships to be stronger at aggregate levels of analysis compared to the individual level, because of lower measurement error at aggregate levels (inter-subjectivity).

Our results provide only partial support for such a perspective. Specifically, of the 12 job conditions-well-being relationships examined, only seven were in the expected positive direction as well as more pronounced at the departmental than at the individual level. This pattern of results was most in evidence for the relationship between well-being, job demands and participation only. Additionally, two of the 12 relationships were positive and stronger at the organizational than at the individual level, these concern participation-well-being relationships.

### Theoretical implications

When integrating all findings, we conclude that our results on the homology of relationships across three levels of analysis were not only mixed but also considerably more varied and complex than we had expected. Overall, our data suggest that the strongest and most consistent stressor-strain relationship is that between job demands and (strain-related) well-being, with the relationship between participation and well-being also emerging as relatively strong and consistent, particularly at supra-individual levels and in relation to task satisfaction as well as job anxiety. It is here that the trickle-down effect, as mentioned in the review paper by Bakker et al. (2023) [6], aligns with our results.

In contrast, most importantly, this study found that the relationships between occupational well-being and both skill variety and task autonomy were much weaker, and the valence tended to vary depending not only on the particular level involved but also on the specific well-being measure under consideration. This indicates that, in our study the three levels in the “trickle down’-cascade of the multi-level chain could at least partially counteract one another. Martin et al. (2016) [21] point out that some job characteristics are more naturally based in resources that occur at the individual level, whereas others are more naturally based in resources at the aggregate level. They emphasize that it is therefore important that each factor is evaluated for its effects at its appropriate level, otherwise there is a mismatch between theory, data, and analysis. Our results reinforce the statements by Martin et al. (2016) [21], especially for skill variety and job autonomy.

This is not the appropriate place for an extensive theoretical analysis of how individual and collective levels of job control and skill variety might combine and interact with one another in impacting on occupational well-being. Our results certainly indicate the need to develop such a theory. And this is certainly not the only study pointing in this direction. Other authors have also begun exploring this area. For example, Wood (2008) [58] researched the simultaneous impact of job control (skill variety and autonomy) and employee voice (participation) on occupational well-being using the UK Work and Employment Relations Survey. Carr & Melizzo (2013) [59], studied how job autonomy and employee voice impact job satisfaction, also building on the WERS dataset. In a highly relevant paper, Tangirala and Ramanujam (2008) [60] hypothesize and confirm how the relationship between job autonomy (personal control) and participation (employee voice) is U-shaped. All three studies mentioned here fail to take multiple levels into account, however.

Relatedly, some reconceptualization may be necessary of job characteristics at various levels of analysis. Whereas for some job characteristics (demands, participation) shared and idiosyncratic situational components may be quite aligned and have consistent effects on occupational well-being, for other job characteristics (skill variety, autonomy) the situation may be more complex, with shared and idiosyncratic components affecting occupational well-being in different ways and directions, partially counteracting or compensating each other in how they influence well-being.

### Practical implications

The results for lack of skill variety/lack of task autonomy partly run counter to the predictions of the theoretical Demand-Control Model and the situationist view on exposure-effect relationships that lies behind it. All in all, it may not be straightforwardly possible to argue for homology of relationships between all types of job control dimensions and occupational well-being across measurement levels. For practice, this implies that one might need to investigate both individual and supra-individual levels separately. For job demands and participation, our results are more promising. Here, we find the best evidence for homology of relationships with occupational well-being from one level of analysis to the next.

Where policy researchers and consultants are using measures similar to our own, we would advise to be careful in interpreting aggregate measures of well-being. Instead, in research of stressor-strain relationships, it is advisable to complement individual-level analysis of relationships with cross-level analysis relating, for example, aggregate job characteristics to individual well-being.

Our results do also have some implications for widely used national or international benchmark systems of job characteristics and occupational well-being (including job satisfaction). In the USA we could mention the Gallup benchmark as a well-known example. Another, internationally recognized system is that of Great Place to Work. Equivalents of these systems exist in other countries [61]. The implication of our study is that aggregate-level interpretations from these data and systems, may build on assumptions that are not necessarily empirically substantiated for all the variables included. Our study does not point to an easy remedy for this problem. What it does suggest, however, is that rethinking job characteristics and their measures could ultimately lead to practical tools and benchmarks that are more solidly evidence-based. Martin et al. (2016) [21] suggest to rely less heavily on individual surveys and use other types of measures like expert ratings or more objective measures instead. And if surveys are still the method of choice, they advise to be much more aware of incorporating appropriate “referents” in the survey, e.g., use the words “departmental” or “organizational” in survey items that aim at measuring job factors at these indicated levels of aggregation.

### Limitations

In this study only individual-level employee data were collected and no measures were used which were specifically designed for the purpose of assessing shared job conditions or shared well-being. That said, it is important to emphasize that the measures used in this study for measuring individual employee perceptions of work autonomy and skill variety have been used and validated extensively in both practical and academic research. The current study is not aiming to capture group or organizational level constructs, rather it is investigating group and organizational level patterns in how individual employees perceive their work autonomy and skill variety.

The findings of our study should be interpreted with caution due to concerns raised by the ICC1 and ICC2 analyses regarding the appropriateness of aggregating data to the departmental and organizational levels in the first place. The ICC1 results indicate that most of the variance in the variables analyzed was at the individual level, with proportions ranging from 77% to 95%. While some fluctuation in shared variance exists at the departmental and organizational levels for the various job characteristics, the proportion of shared variance at these levels was always higher for job characteristics than for well-being measures. However, these ICC1 values are in line with much of the multi-level literature dealing with cognate areas of research, such as climate, culture, and leadership [55], as well as in line with earlier studies in the area of job characteristics and occupational well-being [28,29]. ICC1 values for well-being measures were notably lower, indicating that only a small fraction of the variance (2–4%) was attributable to supra-individual levels, but this is also consistent with earlier research [28,29,38,40]. The levels of ICC2 were mostly adequate, but at the departmental level ICC2 was rather low for the occupational well-being indicators. Caution is necessary in interpreting this part of our results.

Although large, the number of organizations covered in the current study is still relatively small compared to the number of work units and individuals investigated. Thus, the generalizability of our findings at the organizational level should be considered with some caution. In interpreting these results, we also have to bear in mind that the Dutch workforce is highly educated and that the institutional context of labor is quite different from that in other countries [62]. In the Dutch setting, job control may already be established at relatively high levels for large parts of the workforce. For this reason, it is important not to generalize the findings from this study to other countries without proper investigation. There is a need for replication of our findings in other countries.

All measures in this study are based on self-reports, thus making the study vulnerable to possible criticism of common method variance [63]. Spector (2006) [64] however, has argued that calculating correlations among cross-sectional survey scales does not automatically produce a substantial baseline level of correlations, not even in large samples. In this study, the bivariate correlations between job demands and lack of skill variety/lack of participation, are only −.09 and .10 respectively at the individual level. If all self-report measures in this study were systematically biased upwards by individual differences like tendency towards social desirability, negative affectivity, and acquiescence, certainly a much higher baseline value for these correlations might have been expected.

Finally, our results may have been influenced by specific characteristics of our sample in terms of department/organization size and branches of industry. On average, departments contained 11,3 individual respondents, and organizations contained 4,6 departments and 52,1 individual respondents. The average unit size in our study can be characterized as small to medium sized departments/organizations. In a sample containing mostly large departments/large organizations or conversely mostly small departments/small organizations, results might have been different. It is important to replicate our results for samples of different departmental/organizational sizes, as well as different branches of industry.

### Future research avenues

Our study points towards the need to disentangle how various types of variables that pertain to job control and job skills, jointly impact well-being across multiple levels of analysis. This requires progress in terms of concepts, measures as well as theory. In a recent paper Nijstad et al. (2025) [65] provide some useful ideas on how to take all these areas further when considering team research. Here, we conclude with some first ideas for occupational health psychology research.

Conceptually, we think our current results can be interpreted and understood better, when one considers group-level and individual-level components in the survey measures of skill variety and job control as related but different constructs. Although supra-individual components can be interpreted as external, collective environmental attributes, the individual components should not be considered only as “error”. Instead, the individual components might reflect intra-group or social sense-making phenomena (like LMX, I-deals, or social comparisons), which relate to true, actual differences in the idiosyncratic situations of individual workers regarding decision latitude within the same unit or organization. For skill variety and job control, a “composite” model of conceptualizing would seem to apply. Such a “composite” model, combining shared and idiosyncratic situational elements of job characteristics, fits with some earlier attempts at conceptually rethinking job characteristics and their effects [17]. Daniels (2006) [17] pointed out that job characteristics can be latent, perceived, or enacted. The shared component that we mentioned above corresponds to the “latent” category. Any worker in a unit would be similarly affected by a given, similar level of exposure. The “enacted” element captures that, to some extent, exposure is individually crafted by individual workers within a unit, even when the shared level of exposure is similar. This corresponds to the idiosyncratic element we mentioned above.

In terms of measurement, we concur with Morrison et al. (2003) [29] that, especially for the purpose of comparing work units and organizations in terms of occupational well-being, it would be necessary to move towards measures using the unit and/or the organization as the referent for the question, rather than the individual. Chan (1998) [8] and Mason and Griffin (2002) [30], also draw attention to the importance of choosing the right referent in survey items, in connection to the level of the construct to be measured. Whereas in the current study we have used an indirect referencing approach, deducing the work unit/organization level score from statements by individuals about their individual job characteristics and well-being, our results suggest that it would be desirable to try out a direct referencing approach or an objective measurement approach at the work unit/organization level, especially for job skills and job control. In a recent multilevel, multisource study, Li et al. (2022) [66] showed that objective job characteristics as rated by experts in a taxonomic model of jobs were positively related to how these jobs were perceived by employees.

Finally, when concepts and measures have both been clarified and improved according to multilevel lines, there is still the need for theory on how individual level job skills and job control interact with department/organization level skills and control in impacting occupational well-being. Instead of assuming homology across levels for job control, a more nuanced approach is needed that outlines how skills and control at various levels may combine and interact, building on earlier empirical work [58–60].

## Supporting information

S1 File Appendix 1. Mplus-script. (DOCX)

S2 File Dataset. (CSV)

S3 File Codebook. (XLSX)

## References

[1] Botey Gaude L, Cabrita J, Eiffe F, Gerstenberger B, Ivaškaitė-Tamošiūnė V, Parent-Thirion A, et al. EWCS extraordinary edition 2021. European Foundation for the Improvement of Living and Working Conditions; 2021.

[2] Leka S, Jain A. Health impact of psychosocial hazards at work: Nottingham: the European framework for psychosocial risk management. World Health Organization; 2010.

[3] Bakker AB, Demerouti E. Multiple levels in job demands-resources theory: Implications for employee well-being and performance. In: Diener E, Oishi S, Tay L, editors. Handbook of well-being. 2018.

[4] Biron C, Karanika-Murray M, Cooper CL. Improving organizational interventions for stress and well-being. London/New York: Routledge; 2012.

[5] Parker SK, Van den Broeck A, Holman D. Work design influences: a synthesis of multilevel factors that affect the design of jobs. Acad Manag Ann. 2017;11(1):267–308. doi: 10.5465/annals.2014.0054

[6] Bakker AB, Demerouti E, Sanz-Vergel A. Job demands–resources theory: ten years later. Annu Rev Organ Psychol Organ Behav. 2023;10(1):25–53. doi: 10.1146/annurev-orgpsych-120920-053933

[7] Croon MA, Van Veldhoven MJPM, Peccei RE, Wood S. Researching individual wellbeing and performance in context: multilevel mediational analysis for bathtub models. In: Van Veldhoven MJPM, Peccei RE, editors. Well-being and performance at work: The role of context. Psychology Press; 2015. pp. 129–54.

[8] Chan D. Functional relations among constructs in the same content domain at different levels of analysis: a typology of composition models. J Appl Psychol. 1998;83(2):234–46. doi: 10.1037/0021-9010.83.2.234

[9] Klein KJ, Kozlowski S. Multilevel theory, research, and methods in organizations. San Francisco: Jossey-Bass; 2000.

[10] Bolin M, Marklund S, Bliese P. Organizational impact on psychosocial working conditions. WORK: J Prev Assess Rehab. 2008;30(4):451–9. doi: 10.3233/wor-2008-00715 18725708

[11] Finne LB, Christensen JO, Knardahl S. Psychological and social work factors as predictors of mental distress and positive affect: a prospective, multilevel study. PLoS One. 2016;11(3):e0152220. doi: 10.1371/journal.pone.0152220 27010369 PMC4807036

[12] Morrison DL, Payne RL. Multilevel approaches to stress management. Austr Psychol. 2003;38(2):128–37. doi: 10.1080/00050060310001707127

[13] Van der Doef M, Maes S. The job demand-control(-support) model and psychological well-being: a review of 20 years of empirical research. Work Stress. 1999;13:87–114. doi: 10.1080/026783799296084

[14] de Lange AH, Taris TW, Kompier MAJ, Houtman ILD, Bongers PM. “The very best of the millennium”: longitudinal research and the demand-control-(support) model. J Occup Health Psychol. 2003;8(4):282–305. doi: 10.1037/1076-8998.8.4.282 14570524

[15] Peeters M, De Jonge J, Taris T. An introduction to contemporary work psychology. 2nd ed. Hoboken (NJ): Wiley-Blackwell; 2024.

[16] Xiao Q, Liang X, Liu L, Klarin A, Zhang C. How do work-life balance programmes influence nurses’ psychological well-being? The role of servant leadership and learning goal orientation. J Adv Nurs. 2023;79(7):2720–31. doi: 10.1111/jan.15654 36971248

[17] Daniels K. Rethinking job characteristics in work stress research. Hum Relat. 2006;59(3):267–90. doi: 10.1177/0018726706064171

[18] Wegman LA, Hoffman BJ, Carter NT, Twenge JM, Guenole N. placing job characteristics in context: cross-temporal meta-analysis of changes in job characteristics since 1975. J Manag. 2016;44(1):352–86. doi: 10.1177/0149206316654545

[19] Karasek RA. Job demands, job decision latitude, and mental strain: implications for job redesign. Admin Sci Q. 1979;24:285–308.

[20] Spector PE. A consideration of the validity and meaning of self-report measures of job conditions. Int Rev Ind Organ Psychol. 1992;7:123–51.

[21] Martin A, Karanika-Murray M, Biron C, Sanderson K. The psychosocial work environment, employee mental health and organizational interventions: improving research and practice by taking a multilevel approach. Stress Health. 2016;32(3):201–15. doi: 10.1002/smi.2593 25044861

[22] Semmer N, Zapf D, Greif S. “Shared job strain”: a new approach for assessing the validity of job stress measurements. J Occup Organ Psychol. 1996;69:293–310. doi: 10.5271/sjweh.1056

[23] Härenstam A. Organizational approach to studies of job demands, control and health. Scand J Work Environ Health. 2008;Suppl 6:144–9.

[24] Kristensen Tage S. The demand‐control‐support model: Methodological challenges for future research. Stress Med. 1995;11(1):17–26. doi: 10.1002/smi.2460110104

[25] Kristensen TS. Job stress and cardiovascular disease: a theoretic critical review. J Occup Health Psychol. 1996;1(3):246–60. doi: 10.1037//1076-8998.1.3.246 9547050

[26] Ganster DC, Rosen CC. Work stress and employee health: a multidisciplinary review. J Manag. 2013;39(5):1085–122. doi: 10.1177/0149206313475815

[27] Karasek RA, Theorell T. Healthy work: Stress, productivity, and the reconstruction of working life. New York: Basic Books; 1990.

[28] van Veldhoven M, Broersen S. Measurement quality and validity of the “need for recovery scale”. Occup Environ Med. 2003;60 Suppl 1(Suppl 1):i3–9. doi: 10.1136/oem.60.suppl_1.i3 12782740 PMC1765728

[29] Morrison D, Payne RL, Wall TD. Is job a viable unit of analysis? A multilevel analysis of demand-control-support models. J Occup Health Psychol. 2003;8(3):209–19. doi: 10.1037/1076-8998.8.3.209 12872958

[30] Hammer TH, Saksvik PØ, Nytrø K, Torvatn H, Bayazit M. Expanding the psychosocial work environment: workplace norms and work-family conflict as correlates of stress and health. J Occup Health Psychol. 2004;9(1):83–97. doi: 10.1037/1076-8998.9.1.83 14700459

[31] Härenstam A, Bejerot E, Leijon O, Schéele P, Waldenström K, The MOA Research Group. Multilevel analyses of organizational change and working conditions in public and private sector. Eur J Work Organ Psychol. 2004;13(3):305–43. doi: 10.1080/13594320444000119

[32] George JM. Personality, affect, and behavior in groups. J Appl Psychol. 1990;75(2):107–16. doi: 10.1037/0021-9010.75.2.107

[33] Mason CM, Griffin MA. Group task satisfaction: applying the construct of job satisfaction to groups. Small Group Res. 2002;33(3):271–312. doi: 10.1177/10496402033003001

[34] Cole MS, Bruch H, Vogel B. Energy at work: a measurement validation and linkage to unit effectiveness. J Organ Behav. 2011;33(4):445–67. doi: 10.1002/job.759

[35] Costa PL, Passos AM, Bakker AB. Team work engagement: a model of emergence. J Occup Organ Psychol. 2014;87(2):414–36. doi: 10.1111/joop.12057

[36] Urien B, Rico R, Demerouti E, Bakker AB. an emergence model of team burnout. Rev Psicol Trab Organ. 2021;37(3):175–86. doi: 10.5093/jwop2021a17

[37] Söderfeldt B, Söderfeldt M, Jones K, O’Campo P, Muntaner C, Ohlson CG, et al. Does organization matter? A multilevel analysis of the demand-control model applied to human services. Soc Sci Med. 1997;44(4):527–34. doi: 10.1016/s0277-9536(96)00179-7 9015887

[38] Marchand A, Demers A, Durand P. Social structures, agent personality and workers’ mental health: a longitudinal analysis of the specific role of occupation and of workplace constraints-resources on psychological distress in the Canadian workforce. Hum Relat. 2006;59(7):875–901. doi: 10.1177/0018726706067595

[39] Snijders TAB, Bosker RJ. Multilevel analysis: An introduction to basic and advanced multilevel modeling. Thousand Oaks, CA: SAGE; 1999.

[40] Veldhoven M van, Jonge J de, Broersen S, Kompier M, Meijman T. Specific relationships between psychosocial job conditions and job-related stress: a three-level analytic approach. Work Stress. 2002;16(3):207–28. doi: 10.1080/02678370210166399

[41] Van Veldhoven M, Meijman TF. Het meten van psychosociale arbeidsbelasting met een vragenlijst: de vragenlijst beleving en beoordeling van de arbeid (VBBA) [The measurement of psychosocial job demands with a questionnaire: the questionnaire on the experience and evaluation of work (QEEW)]. Amsterdam: Nederlands Instituut voor Arbeidsomstandigheden; 1994.

[42] Warr P. The measurement of well‐being and other aspects of mental health. J Occup Psychol. 1990;63(3):193–210. doi: 10.1111/j.2044-8325.1990.tb00521.x

[43] Karasek RA. Job Content Questionnaire. Los Angeles: Department of Industrial and Systems Engineering, University of Southern California; 1985.

[44] Hackman JR, Oldham GR. Development of the Job Diagnostic Survey. J Appl Psychol. 1975;60(2):159–70. doi: 10.1037/h0076546

[45] Parker S, Wall T. Job and work design: Organizing work to promote well-being and effectiveness. Thousand Oaks, CA: SAGE; 1998.

[46] Wanous JP, Reichers AE, Hudy MJ. Overall job satisfaction: how good are single-item measures? J Appl Psychol. 1997;82(2):247–52. doi: 10.1037/0021-9010.82.2.247 9109282

[47] van Saane N, Sluiter JK, Verbeek JHAM, Frings-Dresen MHW. Reliability and validity of instruments measuring job satisfaction--a systematic review. Occup Med (Lond). 2003;53(3):191–200. doi: 10.1093/occmed/kqg038 12724553

[48] Sonnentag S, Zijlstra FRH. Job characteristics and off-job activities as predictors of need for recovery, well-being, and fatigue. J Appl Psychol. 2006;91(2):330–50. doi: 10.1037/0021-9010.91.2.330 16551187

[49] Sevastos P, Smith L, Cordery JL. Evidence on the reliability and construct validity of Warr’s (1990) well‐being and mental health measures. J Occupat Organ Psychol. 1992;65(1):33–49. doi: 10.1111/j.2044-8325.1992.tb00482.x

[50] Lüdtke O, Marsh HW, Robitzsch A, Trautwein U, Asparouhov T, Muthén B. The multilevel latent covariate model: a new, more reliable approach to group-level effects in contextual studies. Psychol Methods. 2008;13(3):203–29. doi: 10.1037/a0012869 18778152

[51] Muthén B. Multilevel covariance structure analysis. Sociol Methods Res. 1994;22(3):376–98. doi: 10.1177/004912419402200300

[52] Muthén B. Moments of the censored and truncated bivariate normal distribution. Br J Math Stat Psychol. 1990;43(1):131–43. doi: 10.1111/j.2044-8317.1990.tb00930.x

[53] Muthén BO. Latent Variable Modeling in Heterogeneous Populations. Psychometrika. 1989;54(4):557–85. doi: 10.1007/bf02296397

[54] Muthén LK, Muthén BO. Mplus: Statistical Analysis with Latent Variables: User’s Guide. 8 ed. Los Angeles, CA: Authors; 2017.

[55] Bliese PD. Within-group agreement, non-independence, and reliability: implications for data aggregation and analysis. In: Klein KJ, Kozlowski S, editors. Multilevel theory, research, and methods in organizations. San Francisco: Jossey-Bass; 2000. pp. 349–81.

[56] de Jonge J, Kompier MAJ. A Critical examination of the demand-control-support model from a work psychological perspective. Int J Stress Manag. 1997;4(4):235–58. doi: 10.1023/b:ijsm.0000008152.85798.90

[57] Cohen J. Statistical power analysis for the behavioral sciences. 2nd ed. Hillsdale, NJ: Lawrence Erlbaum; 1988.

[58] Wood S. Job characteristics, employee voice and well‐being in Britain. Ind Relat J. 2008;39(2):153–68. doi: 10.1111/j.1468-2338.2007.00482.x

[59] Carr MD, Mellizo P. The relative effect of voice, autonomy, and the wage on satisfaction with work. Int J Hum Resour Manag. 2013;24(6):1186–201. doi: 10.1080/09585192.2012.706818

[60] Tangirala S, Ramanujam R. Employee silence on critical work issues: the cross level effects of procedural justice climate. Personnel Psychol. 2008;61(1):37–68. doi: 10.1111/j.1744-6570.2008.00105.x

[61] Dollard M, Skinner N, Tuckey MR, Bailey T. National surveillance of psychosocial risk factors in the workplace: an international overview. Work Stress. 2007;21(1):1–29. doi: 10.1080/02678370701254082

[62] Boselie P, Paauwe J, Jansen P. Human resource management and performance: lessons from the Netherlands. Int J Human Resour Manag. 2001;12(7):1107–25.

[63] Podsakoff PM, MacKenzie SB, Lee J-Y, Podsakoff NP. Common method biases in behavioral research: a critical review of the literature and recommended remedies. J Appl Psychol. 2003;88(5):879–903. doi: 10.1037/0021-9010.88.5.879 14516251

[64] Spector PE. Method variance in organizational research: truth or urban legend? Organ Res Methods. 2006;9(2):221–32. doi: 10.1177/1094428105284955

[65] Nijstad BA, Homan AC, Heerdink MW, van Kleef GA. Data aggregation in team research: theoretical considerations and practical recommendations. Organ Psychol Rev. 2025. doi: 0.1177/204138662513330

[66] Li Y, Tuckey MR, Bakker A, Chen PY, Dollard MF. Linking objective and subjective job demands and resources in the JD-R model: a multilevel design. Work Stress. 2022;37(1):27–54. doi: 10.1080/02678373.2022.2028319

