# Supplementary material for: Relationships between job characteristics and occupational well-being: Are they similar across levels of analysis?
Source: PLoS One. 2025 Jul 24;20(7):e0328508. doi: 10.1371/journal.pone.0328508 (PMC12289054; doi:10.1371/journal.pone.0328508)
Supplement: S1 File — (DOCX) [file pone.0328508.s001.docx]

**S1Appendix I Mplus syntax for three level homology:**

VARIABLE:

NAMES = Indn Groupn Orgn

jobdem lackskill

lackauton lackpart lacksat

nfr anxiety;

USEVARIABLES ARE Groupn Orgn

jobdem lackskill

lackauton lackpart

lacksat

nfr anxiety;

!Observations are clustered in Groups in Organizations

CLUSTER = Orgn Groupn;

!There are no missing values in the data

!MISSING ARE ALL (999);

ANALYSIS:

!Three-level multilevel with observations in groups in organizations

TYPE = THREELEVEL;

!Use Bayesian estimation

ESTIMATOR = BAYES;

!# of processors to use

PROCESSORS = 2;

!Number of iterations of the sampler

BITERATIONS = (2000);

MODEL:

! The within model

%WITHIN%

jobdem WITH lackskill lackauton lackpart;

lackskill WITH lackauton lackpart;

lackauton WITH lackpart;

lacksat ON jobdem lackskill lackauton lackpart;

nfr ON jobdem lackskill lackauton lackpart;

anxiety ON jobdem lackskill lackauton lackpart;

! The group level model

%BETWEEN Groupn%

jobdem WITH lackskill lackauton lackpart;

lackskill WITH lackauton lackpart;

lackauton WITH lackpart;

lacksat ON jobdem lackskill lackauton lackpart;

nfr ON jobdem lackskill lackauton lackpart;

anxiety ON jobdem lackskill lackauton lackpart;

! The organization level model

%BETWEEN Orgn%

jobdem WITH lackskill lackauton lackpart;

lackskill WITH lackauton lackpart;

lackauton WITH lackpart;

lacksat ON jobdem lackskill lackauton lackpart;

nfr ON jobdem lackskill lackauton lackpart;

anxiety ON jobdem lackskill lackauton lackpart;

OUTPUT: TECH1 TECH8 STANDARDIZED;

PLOT: TYPE = PLOT3;
